# Significant early and long-term improvement of neuropsychiatry symptomatology in HCV-infected patients after viral eradication with DAA

**DOI:** 10.1192/j.eurpsy.2022.616

**Published:** 2022-09-01

**Authors:** R. Martin-Santos, C. Bartrés, L. Nacar, R. Navinés, M. Cavero, S. Lens, S. Rodriguez-Tajes, J.C. Pariante, I. Horrillo, E. Muñoz-Moreno, N. Bargallo, L. Capuron, J. Meana, X. Forns, Z. Mariño

**Affiliations:** 1 Hospital Clínic,, 1department Of Psychiatry And Psychology, Barcelona, Spain; 2 Hospital Clínic,, UB, IDIBAPS, CIBERSAM, Liver Unit, Barcelona, Spain; 3 Hospital Clínic,, UB, IDIBAPS, CIBERSAM, 1department Of Psychiatry And Psychology, Barcelona, Spain; 4 Hospital Clinic, IDIBAPS, Neuroradiology Section, Barcelona, Spain; 5 Universidad del Pais Vasco, Pharmacology, Bilbao, Spain; 6 INRA, Universite Bordeaux, Laboratory Of Nutrition And Integrative Neurobiology, Bordeaux, France

**Keywords:** mood, treatment response, viral hepatitis, cognition

## Abstract

**Introduction:**

Chronic Hepatitis C infection is considered a systemic disease with extrahepatic manifestations, mainly neuropsychiatric symptoms^,^ which is associated with a chronic low-grade inflammatory state^.^ Hepatitis C virus (HCV) eradication is currently achieved in >98% of cases with oral direct-acting antivirals (DAA).

**Objectives:**

To study potential clinical neuropsychiatric changes (mood, cognition, sleep, gastrointestinal, sickness, and motion) in HCV-infected patients after HCV eradication with DAA.

**Methods:**

Design: Cohort study. Subjects: 37 HCV-infected patients, aged<55 years old, with non-advanced liver disease receiving DAA; free of current mental disorder. 24 healthy controls were included at baseline. Assessment: -Baseline (BL) (socio-demographic and clinical variables, MINI-DSM-IV, and Neurotoxicity Scale (NRS), (mood, cognitive, sleep, gastrointestinal, sickness and motor dimensions). Follow-up: End-of-treatment, 12weeks-after and 48weeks-after DAA: NRS. Analysis: Descriptive and bivariate non-parametrical analysis.

**Results:**

NRS total score and dimensions where different between cases and controls (.000) at baseline. NRS total score (.000) and mood (.000), cognition (.000), sleep (.002), gastrointestinal (.017), and sickness (.003), except motor dimension score (.130) showed significant longitudinal improvement.

**Conclusions:**

HCV-infected patients with mild liver disease presented significantly worse scores for neurotoxicity symptomatology in all dimensions compared to healthy individuals. After HCV eradication with DAA, both at short and long follow-up a significant improvement of the NRS total score and each of the dimensions (except motor) were observed. However, they did not reach the values of healthy individuals, suggesting a not complete neuropsychiatric restoration in the period studied. Grant: ICIII-FIS:PI17/02297.(One way to make Europe) (RMS) and Gilead Fellowship-GLD17/00273 (ZM); and the support of SGR17/1798 (RMS)

**Disclosure:**

No significant relationships.

